# Thyroxine (T4) may promote re-epithelialisation and angiogenesis in wounded human skin *ex vivo*

**DOI:** 10.1371/journal.pone.0212659

**Published:** 2019-03-29

**Authors:** Guo-You Zhang, Ewan A. Langan, Natalia T. Meier, Wolfgang Funk, Frank Siemers, Ralf Paus

**Affiliations:** 1 Department of Plastic and Reconstructive Surgery, Shanghai Ninth People’s Hospital, Shanghai Jiaotong University School of Medicine, Shanghai, China; 2 Department of Dermatology, University of Lübeck, Lübeck, Germany; 3 Centre for Dermatology Research, University of Manchester, and NIHR Manchester Biomedical Research Centre, Manchester, United Kingdom; 4 Department of Pathology, University of Lübeck, Lübeck, Germany; 5 Klinik Dr. Funk, Munich, Germany; 6 Department of Plastic and Hand Surgery, BG Klinikum Bergmannstrost, Halle/Salle, Germany; 7 Department of Dermatology & Cutaneous Surgery, University of Miami Miller School of Medicine, Miami, Florida, United States of America; 8 Centre for Dermatology Research, University of Manchester, and NIHR Manchester Biomedical Research Centre, Manchester, United Kingdom; University of Pisa, ITALY

## Abstract

There is a pressing need for improved preclinical model systems in which to study human skin wound healing. Here, we report the development and application of a serum-free full thickness human skin wound healing model. Not only can re-epithelialization (epidermal repair) and angiogenesis be studied in this simple and instructive model, but the model can also be used to identify clinically relevant wound-healing promoting agents, and to dissect underlying candidate mechanisms of action in the target tissue. We present preliminary *ex vivo* data to suggest that Thyroxine (T4), which reportedly promotes skin wound healing in rodents *in vivo*, may promote key features of human skin wound healing. Namely, T4 stimulates re-epithelialisation and angiogenesis, and modulates both wound healing-associated epidermal keratin expression and energy metabolism in experimentally wound human skin. Functionally, the wound healing-promoting effects of T4 are at least partially mediated via fibroblast growth factor/fibroblast growth factor receptor-mediated signalling, since they could be significantly antagonized by bFGF-neutralizing antibody. Thus, this pragmatic, easy-to-use full-thickness human skin wound healing model provides a useful preclinical research tool in the search for clinically relevant candidate wound healing-promoting agents. These *ex vivo* data encourage further pre-clinical testing of topical T4 as a cost-efficient, novel agent in the management of chronic human skin wounds.

## Introduction

Retarded healing of human skin wounds, which may result in ulceration, represents an increasing, global healthcare and quality-of-life challenge, especially in the context of an aging population [1–5]. Indeed, there is a pressing need for the identification of effective, safe and cost-efficient wound healing promoters which can be introduced into clinical practice [6]. In order to meet this need, it is critical to have simple and pragmatic, predictive model systems in which new candidate promoters of human skin wound healing can be instructively studied at the preclinical level [7, 8]. Though modelling wound healing in so-called 3D skin “equivalent” culture systems is informative [9], such systems usually lack skin appendages, immune cells, for example macrophages and mast cells, and other resident skin cells, and do not reflect the tissue tension characteristics of human skin, all of which are already known to significantly modulate cutaneous would healing [10–16]. Therefore, we and others have advocated the use of experimentally wounded full-thickness human skin *ex vivo*, ideally under defined, serum-free organ culture conditions, as well as the systematic testing of agents that have already been licensed for clinical use [7].

Thyroid hormones (THs) are of special interest in this context, since human skin and hair follicles are classical TH target organs [17–27], while thyroid diseases affect skin structure and function on multiple levels [28–30]. For example, L-thyroxine (T4) promotes human hair growth [19] and stimulates wound healing *in vivo* in rats [31] and mice [32]. Moreover, T4 operates as the chief endocrine control of amphibian metamorphosis [33], suggesting that it can act as a powerful morphogen. In addition, T4 is one of the most frequently administered hormones in clinical medicine, where it has been in extensive use for decades, its toxicology is very well-examined [34], and it is relatively inexpensive. Yet, the potential clinical utility of T4 in a dermatological setting, namely as a candidate wound healing promoter, is yet to be fully explored [30].

Given the reported wound healing-promoting properties of T4 in rodents [31, 32] and the strong interdependence of cutaneous wound healing, hair follicle (HF) cycling, HF neogenesis and skin stem cell activities [35–37], we hypothesized that T4 may also promote human skin wound healing. In order to probe whether T4 directly impacted on human skin wound healing, i.e. in the absence of other systemic/endocrine inputs, we tested our hypothesis in serum-free organ culture of full-thickness human skin [38] that had been experimentally wounded, using a “punch-in-a-punch” design [7, 39].

**Fig 1:**
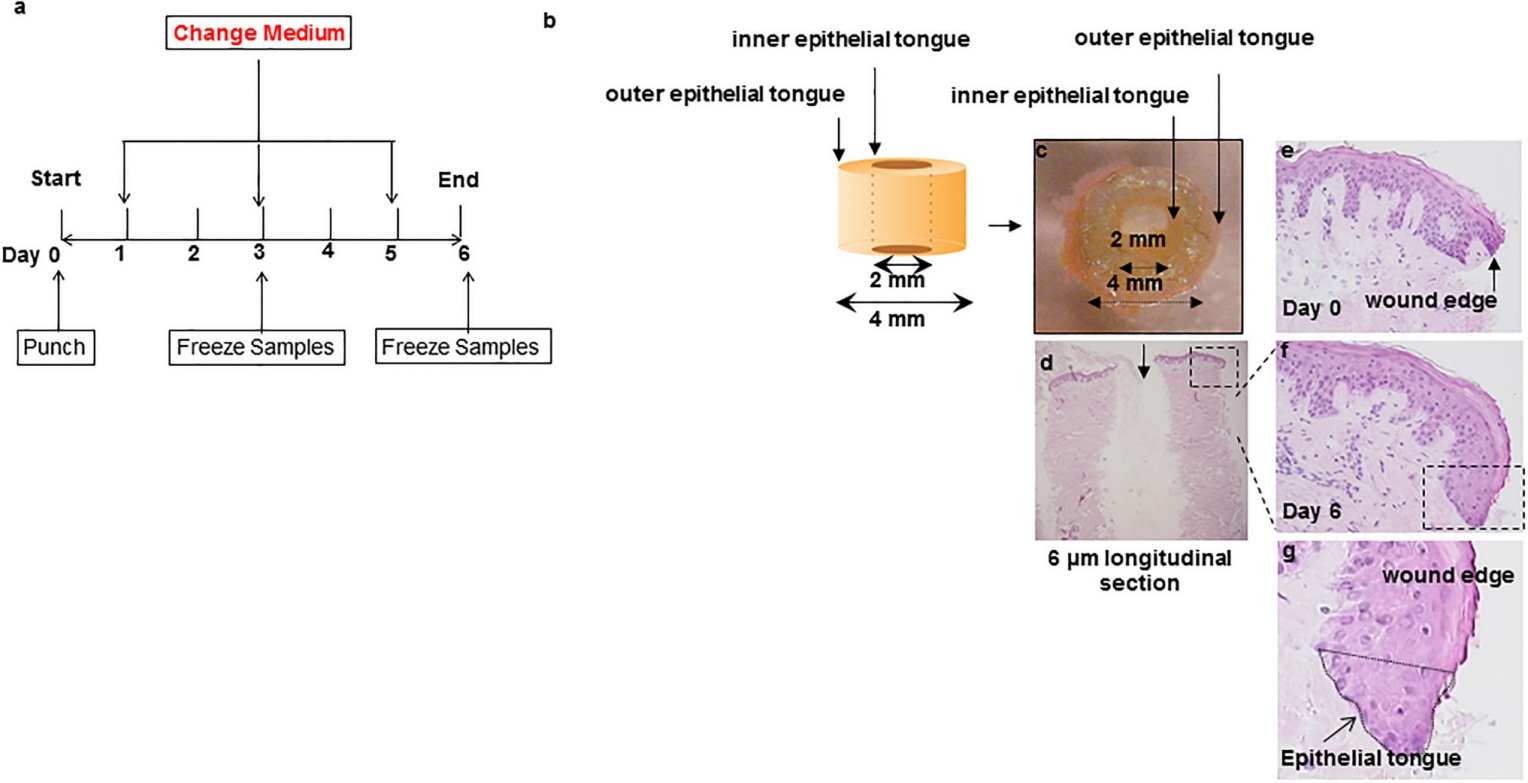
Establishment of human wound healing assay and testing of effects of T4 on keratinocyte migration during epidermal repair of experimentally wound human skin. (**a**) A schematic diagram of human wound healing culture model assay. A schematic diagram (**b**) and example of a wound punch sample (**c**) and longitudinal section (**d**). Sections from day 0 and day 6 of culture (vehicle control) are shown (**e-g**). The regenerative phenomenon is highlighted in (**f**). Magnification of marked area in (**f**) is shown in (**g**).

Recognizing that re-epithelialisation and angiogenesis are key determinants of physiological cutaneous wound healing [10, 37, 40–42] we primarily assessed T4 effects on the regenerated epidermis (‘epithelial tongues’ [ET]) at the inner and outer edges of wounded skin fragments [7, 43] and on intradermal angiogenesis (see Fig 1). Re-epithelialisation can be instructively quantified by planimetric measurement of both the mean length (as an indicator of keratinocyte migration) and the combined areas of the inner and outer ET (as an indicator of total epithelial regeneration) [7] (Fig 1a–1g).

Evidence for angiogenesis was assessed by quantitative CD31 immunohistomorphometry, namely CD31 immunoreactivity (IR) and the number of CD31 positive cells and cross-sectional lumina [44]. In addition, we measured the IR of basic fibroblast growth factor (bFGF, FGF2) since this pro-angiogenic growth factor is up-regulated by T4 [45, 46] and is known to play a key role as positive regulator of wound healing [47–52]. Finally, we also assessed how T4 impacts on the key wound healing associated keratin 6 (CK6) [7, 53, 54].

## Material and methods

Williams’ E medium (Biochrom, Cambridge, UK) supplemented with 2 mmol/liter L-glutamine (Invitrogen, Paisley, UK), 10 ng/ml hydrocortisone (Sigma-Aldrich, Taufkirchen, Germany), 10 μg/ml insulin (Sigma), and antibiotic mixture (100 U/ml Penicillin, 10 microg/ml Streptomycin) (Sigma-Aldrich, Taufkirchen, Germany) [19, 55]. Thyroxine (T4) was obtained from Sigma. Goat polyclonal bFGF-neutralizing antibody [45] was obtained from R&D systems, Minneapolis, MN (anti-FGF basic Antibody, AB-233-NA).

### Skin samples

Human scalp or corporal skin samples were obtained from patients undergoing plastic or reconstructive surgical procedures with informed consent and Institutional Research Ethics Committee permission (University of Luebeck: 06–109). The study was conducted according to The Helsinki Declaration of 1975 (revised 1983). Our study included samples from 6 patients aged from 26–67 years (average 52.6 years) (details, see Table 1).

**Table 1:** Characteristics of patients included in this study.

Patient	Age (years)	Sex	Location
Patient 1^a^	67	F	Temporal
Patient 2^a^	42	F	Breast
Patient 3^a^	59	F	Forearm
Patient 4^b^	61	F	Face
Patient 5^b^	61	F	Temporal
Patient 6^b^	26	M	Buttock

^a^: used for T4 experiment analysis;

^b^: used for inhibitory bFGF antibody treatment experiments

### Human skin wound healing organ culture model

The human skin wound healing assay modified based on the previously published “punch-in-a-punch” design [7, 8, 39] with the notable difference being that full-thickness (including subcutaneous fat) adult human skin was used and cultured in serum-free medium [38] (Fig 1). In the pilot study reported here, one to two “punches” were obtained for each experimental condition (control, T4 10, 100 or 1000nM) from each patient at the beginning of each culture and snap frozen at day 3 or 6 depending on the experimental group. Samples were frozen immediately for analysis (day 0) or transferred to six-well plates containing supplemented Williams E culture medium [38]. Each well contained 1–2 skin punches in 3 ml of medium. Skin samples were left untreated (“equilibration period”) for the first 24 hours of the culture period. Then the medium was replaced for all samples; in the test conditions T4 was tested at concentrations ranging from 10 to 1000 nM, based on our previous human HF organ culture study [19]. Control and T4 supplemented culture medium were changed every 2 days and sample freezing was performed as per the culture protocol (Fig 1). Human skin fragments were embedded in Shandon Cryomatrix (Thermo Fisher Scientific; Waltham, MA, USA) before longitudinal cryosections (6μm) were obtained. Cryosections were stored at -80°C until used.

### Immunohistochemistry, immunofluorescence and quantitative immunohistomorphometry

The antibodies and corresponding detection methods which were used are described in Table 2. For detection of proliferating and apoptotic cells in this system, Ki67/TUNEL quantitative-immunohistomorphometry was performed as described previously [19, 23, 24, 36, 38, 56–58]. For the quantitative evaluation of the double-immunostaining results, DAPI-, Ki67-, or TUNEL-positive cells were counted in defined reference areas (see dotted line) in the newly regenerated epidermis (ETs), (Fig 1). The number of DAPI-positive cells served as “total number of cells”, and the percentage of Ki-67-positive and/or TUNEL positive cells was calculated on this basis to enable comparison between control and test groups.

**Table 2:** Antibodies used for immunohistology.

*Name*	*Host*	*Dilution*	*Method*	*Source*	*Positive control*	*Clone*
**MTCO1**	Mouse	1:50	AEC	Mitosciences, Eugene, OR, USA	Skin epidermis [60, 61]	1D6E1A8
**Keratin 6**	Mouse	1:10	Indirect IF	PROGEN, Heidelberg, Germany	Suprabasal layers of the ORS; suprabasal layers of wounded skin [63, 79]	Ks6.KA12
**bFGF**	Mouse	1:50	Indirect IF	Abcam, Cambridge, UK	Epidermis [59]	ab181
**FGFR1**	Mouse	1:100	Indirect IF	Abcam, Cambridge, UK	Epidermis [59]	ab829
**PCAM (CD31)**	Mouse	1:30	Indirect IF	Dako, Glosturp, Denmark	Dermal microvessels [44]	M0823

MTCO1: cytochrome c oxidase 1; IF: immunofluorescence; TSA: Tyramide Signal Amplification; PECAM: Platelet Endothelial Cell Adhesion Molecule; CTS: connective tissue sheath

Standard haematoxylin and eosin staining was used to determine the new ETs. Cytokeratin 6, CD31, bFGF, and fibroblast growth factor receptor 1 (FGFR1) immunofluorescence were detected using the previously described methods [7, 19, 44, 59]. Mitochondrially encoded cytochrome c oxidase 1 (MTCO1) IR was detected by peroxidase-based avidin-biotin complex immunostaining, without counterstaining with Haematoxylin. A monoclonal antibody that selectively recognizes the mitochondria-specific complex IV subunit of cytochrome oxidase c was employed. [26, 27, 60, 61]. We had previously documented that the intensity of keratinocyte MTCO1 IR *in situ* correlates with the activity of respiratory chain complexes I and IV [26, 27, 60]. Given that the angiogenic effects of T4 may be mediated via upregulation of FGFR [45, 52], we cultured 1–2 “punches” of skin with either 100nM T4 or 100nM T4 plus inhibitory bFGF (8μg/ml) antibody in short-term organ culture for 3 days.

For quantitative immunohistomorphometry [23, 24, 56–58], the IR in the ETs was analysed. Both outer and inner epithelial tongue were analysed, defined as the area from the edge of the stratum corneum where the punch had been placed to the corresponding point in the epidermal basal layer. For the angiogenesis parameters, CD31 IR, the number of CD31 positive nuclei and the number of vessel lumina were determined as previously described [44]. The Image J software (National Institute of Health, Bethesda, MD) was used for evaluation. All the samples were photographed for analysis with a Keyence Biozero-8000 Microscope (Keyence Corporation, Higashi-Nakajima, Osaka, Japan).

### Statistical analysis

All the data are given as mean ± standard error of the mean (SEM). For the quantitative immunohistomorphometry analyses, the IR in the ETs was measured in up to 4 sections for each wounded skin fragment. One-Way ANOVA by appropriate post hoc comparisons was used at single time points, and if the data did not follow a Gaussian distribution, non-parametric tests were applied (i.e. Kruskal-Wallis test). Statistical analysis was carried out by Graphpad prism 5.01 (Graph Pad software, Inc., San Diego, CA, USA), and *p*<0.05 was regarded as significant.

## Results

After wounding, a compact sheet of epidermal keratinocytes (the ETs) began to cover the wound edges in all groups (epiboly phenomenon [62]), as expected [21]. Histochemically, overall skin morphology was well-preserved until and including day 6 of organ culture, during which time no epidermal detachment from the basal membrane was seen, while the number of proliferating or apoptotic keratinocytes in the epidermis and HFs (Ki-67/TUNEL immunofluorescence microscopy) during the entire study window of 6 days was within the expected normal range [26, 38].

**Fig 2:**
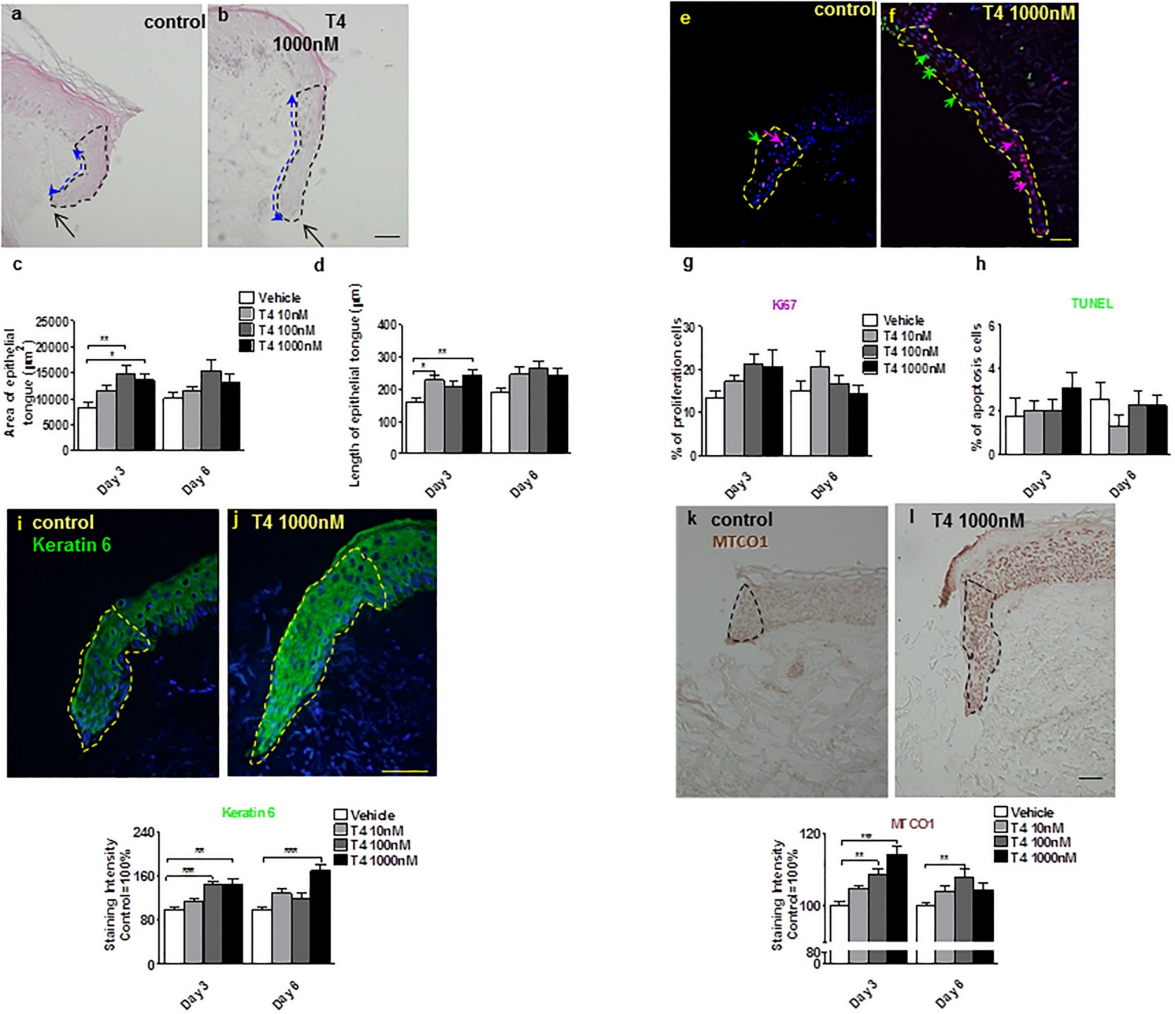
T4 promotes human skin re-epithelialisation. (**a-d**) Haematoxylin and eosin histochemistry: overview of wounded human skin fragment. After 3 days, epithelial tongue areas (blacked dotted line area) (ET) and length (blue dotted line) were significantly greater after treatment with T4 compared to control alone. (**e-h**) Cryosections of control-, or T4-treated human skin were examined by **Ki-67** (red arrow)/**TUNEL** (green arrow) double-labelling [19, 23, 24, 56, 57] (**e, f**). The percentage of positive cells was analyzed in the new ETs (see dotted line). When compared to the control group, more Ki-67 positive cells after 3 day culture with 1000 nM T4 treatment (and more TUNEL positive cells at day 3 in T4 1000 nM treated group were found, although the differences were not statistically significant (**e-h**). (**i, j**) **CK6** expression was significantly upregulated by T4, especially in the 1000 nM treatment group. Green fluorescence staining represented cytokeratin 6 IR in the new wound ET (dotted line area).(**k, l**) **MTCO1** expression was significantly up-regulated by T4. Brown staining represents **MTCO1** IR in the new ET. Staining intensity was measured in a defined reference area (dotted line) and normalized to the control (100%), as for CK6 expression. Number of independent experiments: n = 3 subjects (i.e. 1–2 punches per patient, per treatment group and per time point and at least 8 photomicrographs were analysed per condition); data were pooled since the results trends in all three independent experiments were comparable). *p<0.05, **p<0.01, ***<0.001. Scale bars = 50μm.

Compared to the vehicle control, T4 administered to the culture medium significantly stimulated re-epithelialisation by day 3 after skin wounding in organ culture (Fig 2a–2d). Thereafter, higher concentrations of T4 rather slowed-down ET elongation (possibly due to an inhibition of keratinocyte migration). However, the total ET area (i.e. the mass of regenerated epithelium) was persistently larger in T4-treated compared to vehicle-treated wound skin fragments *ex vivo* (Fig 2a–2d).

Proliferation and apoptosis in the ETs were not significantly modulated by T4 (only high-dose T4 treatment showed a [non-significant] proliferation-stimulatory trend in the ETs at day 3), as measured by quantitative immunohistomorphometry of Ki67+ or terminal deoxynucleotidyl transferase dUTP nick end labelling-positive (TUNEL+) cells in ETs (Fig 2e–2h). This may reflect the well-recognized complexity of T4’s effects on the overall tissue modelling process, which represents a balance of keratinocyte proliferation, apoptosis, differentiation and migration effects [33]. This observation suggests that the re-epithelialisation-promoting effects of T4 primarily result from the stimulation of keratinocyte migration.

T4 also significantly increased expression of the major wound healing-associated keratin, keratin 6 [53, 63] (Fig 2i and 2j), and of the mitochondrial activity protein marker, MTCO1 [60, 61] (Fig 2k and 2l), whose expression is well-correlated with respiratory chain complexes I and IV activity in human epidermis [26, 60, 61].

**Fig 3:**
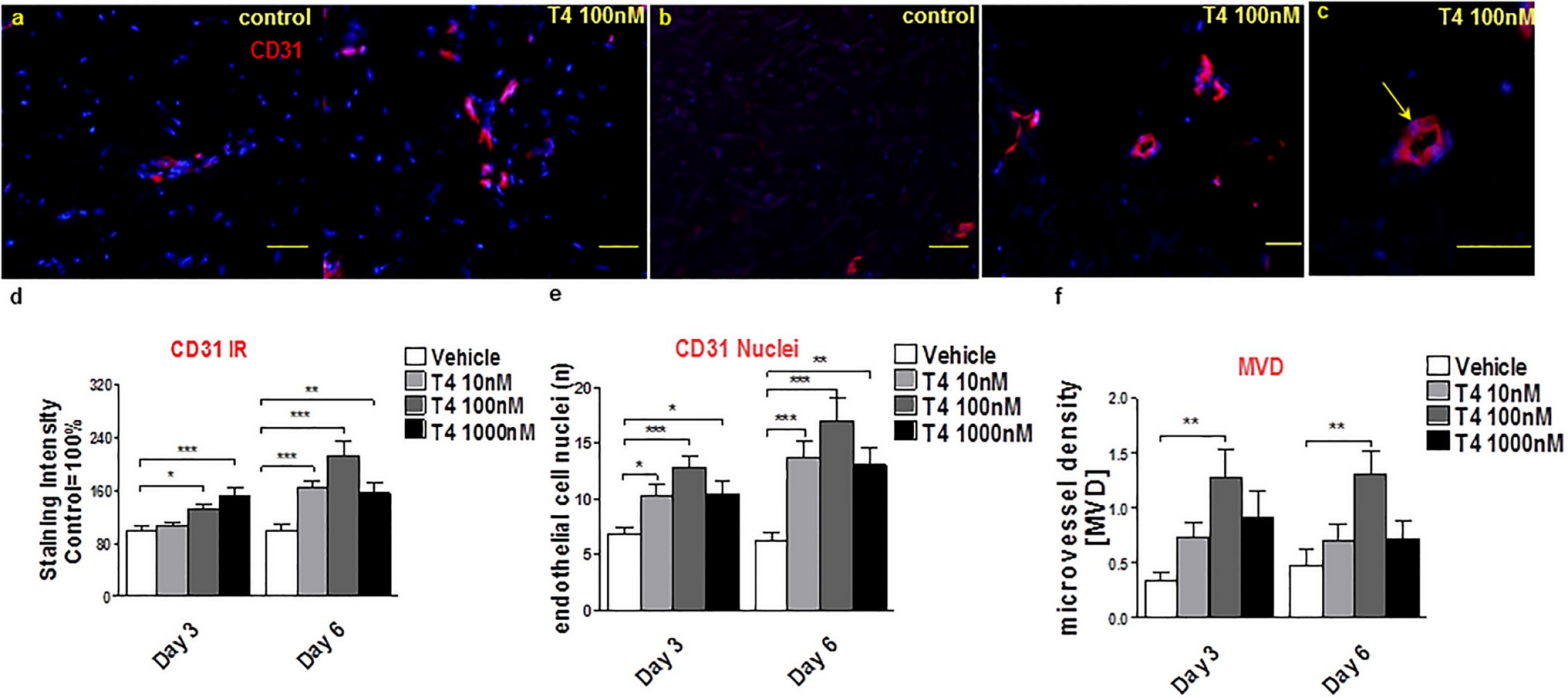
T4 stimulates angiogenesis in wounded human skin. (**a-c**) To analyze angiogenesis, the number of CD31+ cells (red) and of CD31+ blood vessel cross-sections (lumina) (yellow arrow, **c**) per visual field were counted by immunofluorescence microscopy (at least 12 visual fields per skin fragment were evaluated). In addition, the intensity of CD31 IR was measured. Scale bars in **a, b** = 50μm, **c** = 200μm. (**d**) CD31 IR was significantly up-regulated by T4 at days 3 and 6. Immunoreactivity data was normalized to the control data as were (**e, f**) the number of CD31 +ve endothelial cell nuclei (CD31+/DAPI+ cells) and lumina per microscopic field. Number of independent experiments: n = 3 subjects (i.e. 1–2 punches per patient, per treatment group and per time point and at least 8 photomicrographs were analyzed per condition); data were pooled since the results trends in all three independent experiments were comparable). MVD: Microvessel density; ibFGF ab: inhibitory bFGF antibody.

Furthermore, T4 treatment moderately increased CD31 IR, the number of CD31+ positive endothelial cells in T4-treated wounded skin fragments (Fig 3a–3e) and the microvessel density (Fig 3a, 3b and 3f), measured as described previously [44]. This suggests that T4 enhanced angiogenesis in wounded human skin fragments *ex vivo*, despite the fact that these cutaneous blood vessels are non-perfused and may collapse to some degree after surgical skin removal. Whilst the effect was modest, it was statistically significant.

**Fig 4:**
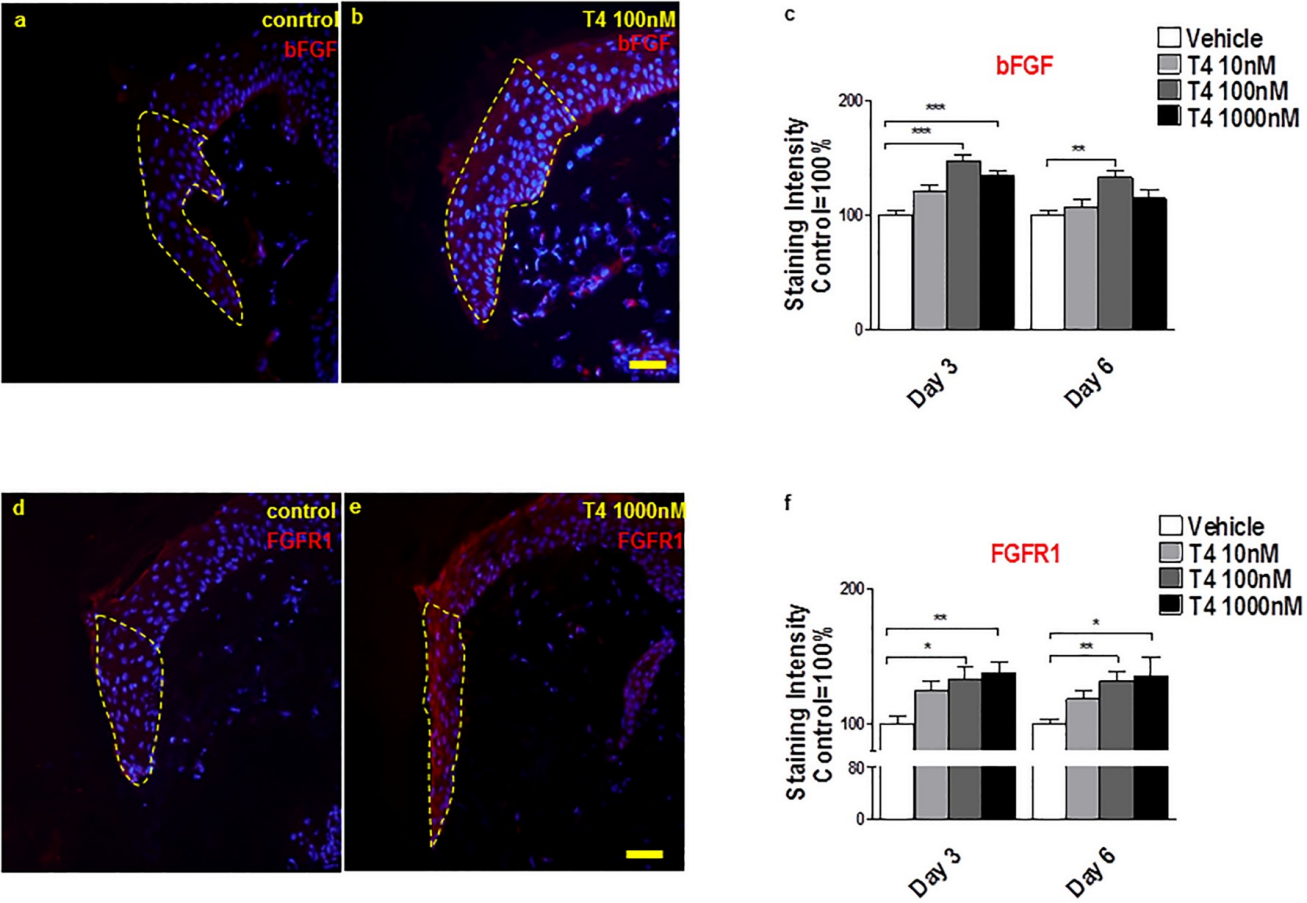
T4 up-regulates bFGF and FGF receptor 1 IR. (**a-c**) bFGF expression was significantly up-regulated by T4 in the epidermis of new epithelial tongues (staining intensity was measured in reference area) at day 3 and day 6 in 100nM T4 condition. Red fluorescence staining represents bFGF IR in the new epithelial tongue and the control IR value was normalized as 100%. (**d-f**) T4 increases FGFR1 expression. Red fluorescence staining represents FGFR1 IR in the new epithelial tongue. Staining intensity was measured in the dotted line reference area and the control IR value was normalized as 100%. One-Way ANOVA by appropriate post hoc comparisons was used. Data represent the mean±SEM of 3 independent experiments and normalized to the control as 100%. 1–2 punches per patient, per treatment group and per time point and at least 8 photomicrographs were analyzed per condition. *P<0.05; **P<0.01; ***P<0.001. Scale bars = 50μm.

Since bFGF is a key pro-angiogenic factor known to be up-regulated by T4 [45, 46], we further examined the role of this growth factor role in T4-stimulated wounded human skin. Indeed, T4 increased bFGF protein IR in the ETs (Fig 4a–4c). Next, FGFR1 IR was assessed, since the proangiogenic actions of T4 may be indirectly mediated at least in part via up regulating FGFR1 expression [64]. In fact, as shown in Fig 4d–4f, FGFR1 was significantly increased in ETs by T4 compared to control.

**Fig 5:**
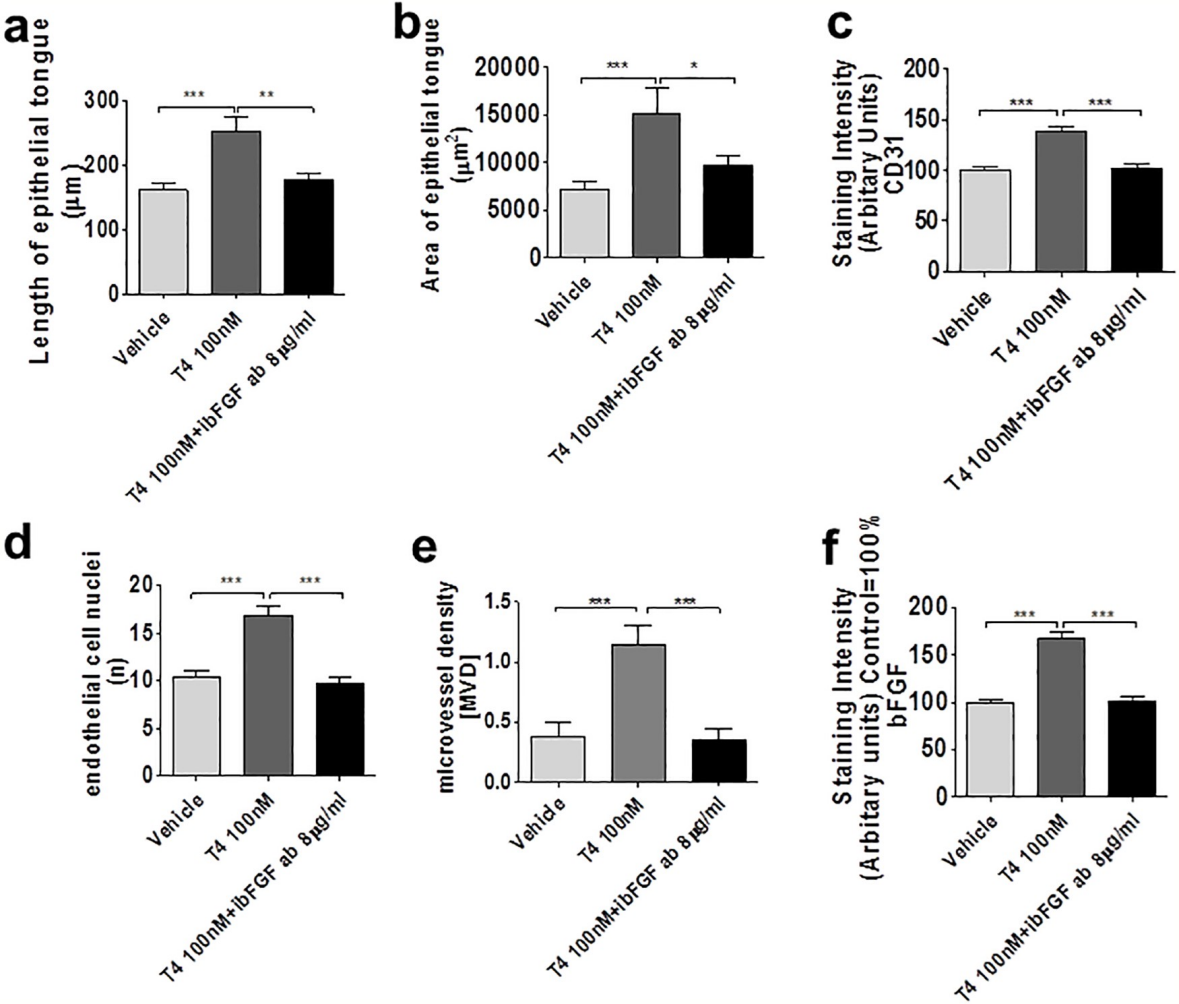
(a-f) T4 mediates its effect at least partially via bFGF. T4 exerts its effect partly through inducing bFGF at day 3. Area and length of the ET (**a**: length of new ET; **b**: area of new ET), intensity of the IR of CD31 (**c**), number of CD31+ cells (**d**), Number of CD31+ lumina (**e**), and intensity of IR of bFGF in new ET (**f**) were significantly increased in the T4-treated test group compared to controls. 1–2 punches per patient, per treatment group and per time point and at least 8 photomicrographs were analyzed per condition. These effects could be abrogated by co-treatment with inhibitory bFGF antibody [45].

Most importantly, co-administration of inhibitory-bFGF antibody [45] counteracted the stimulatory effects of T4 on re-epithelialisation (Fig 5a and 5b), CD31 protein IR (Fig 5c), and number of CD31+ endothelial cells (Fig 5d), microvessel density (Fig 5e) and bFGF expression (Fig 5f). Taken together, this suggests that the wound healing-promoting effects of T4 in organ-cultured human skin are, at least in part, bFGF-dependent.

## Discussion

Here we report how organ-cultured, experimentally wounded human skin can be used to identify clinically relevant wound-healing promoting drugs, and to dissect underlying candidate mechanisms of action. The main advantage of the current model is its use of full-thickness human skin (which is readily available as excess tissue from plastic surgery) in a serum-free setting. Thus, not only the interaction between the epidermis, dermis and subcutaneous tissue is preserved, but the skin appendages, including intact HFs and sweat glands, all of which may play a substantial role in wound healing [37, 65, 66] are also present. In contrast to 3D skin culture models, it is therefore potentially possible to study the influence of the full range of resident skin cell populations on cutaneous wound healing. Future work may utilise the model to carefully study the influence of the HF on wound healing in human skin *ex vivo*. Notably, we show *ex vivo* data to suggest that T4, a widely used, cost efficient drug with well-known toxicology, may promote key features of human skin wound healing.

The model also enables selected skin signalling pathways to be studied, just as in serum-free human HF organ culture [67–69]. Namely, provide preliminary preclinical evidence that the re-epithelialisation- and angiogenesis-promoting effects of T4 in experimentally wounded human skin *ex vivo* primarily are due to the up-regulation of bFGF/FGFR1-mediated signalling in the wounded epithelium.

Of course, it is important to also recognize the limitations of the *ex vivo* model used here. These limitations include, besides the obviously missing functional skin innervation and perfusion, the relatively short time-frame in which wound healing can be studied here. After approximately 6 days, epidermal detachment becomes a prominent feature, preventing long-term application of the model. For this reason we limited the culture time to avoid confounding the data by increasing tissue degeneration effects, and thus focused on early to medium-term interventions to promote wound healing. Another important limitation is that we used skin explants from skin containing either terminal or vellus HFs. Recently studies have confirmed the superior wound healing promoting properties of hair-bearing skin when compared to skin from “hair-free” sites [65]. Whether T4 would exert superior wound healing promoting properties in terminal hair-bearing skin compared to skin dominated by vellus HFs or hair-free palmoplantar skin remains to be clarified. It must also be borne in mind that the thyroid-status of the subjects at the time of surgery was not known.

To consolidate the pilot results reported here, it is important that the current study is repeated both, with skin from an increased number of subjects and in additional wound healing models, including. for example humanized mouse *in vivo* models of wound healing [70] to confirm the positive effect of T4 on wound healing. Such pre-clinical testing is essential given the possible side-effects of topical T4 application in human subjects, included systemic absorption which might lead to a hyperthyroid state, a key consideration as increased T4 serum levels are strictly to be avoided. Reassuringly though, topical T4 may fail to influence circulating T4 levels significantly [71]. Whether the absence of perfusion-derived endocrine signalling *ex vivo* may render organ-cultured skin “functionally” hypothyroid and whether this is compensated for by increased intracutaneous conversion of residual T4 to tri-iodothyronine [19] is unknown.

Caution must be exercised with extrapolating the effects of T4 on human skin healing *ex vivo* to the more complex *in vivo* situation, as the model does not satisfactorily permit the study of the important contributions of platelets, neutrophils and circulating T cells to wound healing [72, 73]; while resident immunocytes like mast cells and macrophages are abundantly present and can be instructively studied *ex vivo* [74–77] Yet, the ex vivo effects of T4 reported here are well in line with its *in vivo* effects reported in rodent wound healing models [22, 31, 32]

Given the overall medical importance of wound healing disorders and the urgency to develop more effective, affordable, and safe wound healing-promoting agents for the treatment of chronic skin ulcers [7, 78]. Our pilot data encourage the preclinical systematic exploration of T4 as a potential wound healing promoter in experimentally wounded human skin, using additional wound healing models [8] as the next step before determining whether T4 is a plausible future skin ulcer therapy.

## Supporting information

S1 Table (Immuno)histochemical evaluations. (XLSX)

## Abbreviations

bFGF: basic fibroblast growth factor
CK: cytokeratin
DAPI: 4’-6-diamidino-2-phenylindole
ET: epithelial tongue
FGFR1: fibroblast growth factor receptor 1
HF: hair follicle
IR: immunoreactivity
T4: thyroxine
TH: thyroid hormones
TUNEL: terminal deoxynucleotidyl transferase dUTP nick end labelling

## References

[1] Ilonzo N, Patel M, Lantis JC 2nd. Managing the Diabetic Foot Ulcer: How Best Practices Fit the Real 2018 United States. Surg Technol Int. 2018;32:49–59. Epub 2018/04/04. . 29611155

[2] Guest JF, Fuller GW, Vowden P. Venous leg ulcer management in clinical practice in the UK: costs and outcomes. Int Wound J. 2018;15(1):29–37. Epub 2017/12/16. 10.1111/iwj.12814 . 29243398 PMC7950152

[3] Coleman S, Smith IL, McGinnis E, Keen J, Muir D, Wilson L, et al Clinical evaluation of a new pressure ulcer risk assessment instrument, the Pressure Ulcer Risk Primary or Secondary Evaluation Tool (PURPOSE T). J Adv Nurs. 2018;74(2):407–24. Epub 2017/08/24. 10.1111/jan.13444 . 28833356 PMC5846883

[4] Xie T, Ye J, Rerkasem K, Mani R. The venous ulcer continues to be a clinical challenge: an update. Burns Trauma. 2018;6:18 Epub 2018/06/27. 10.1186/s41038-018-0119-y . 29942813 PMC6003071

[5] Jeffcoate WJ, Vileikyte L, Boyko EJ, Armstrong DG, Boulton AJM. Current Challenges and Opportunities in the Prevention and Management of Diabetic Foot Ulcers. Diabetes Care. 2018;41(4):645–52. Epub 2018/03/22. 10.2337/dc17-1836 . 29559450

[6] Pastar I, Wong LL, Egger AN, Tomic-Canic M. Descriptive vs mechanistic scientific approach to study wound healing and its inhibition: Is there a value of translational research involving human subjects? Exp Dermatol. 2018;27(5):551–62. Epub 2018/04/17. 10.1111/exd.13663 . 29660181 PMC6374114

[7] Meier NT, Haslam IS, Pattwell DM, Zhang GY, Emelianov V, Paredes R, et al Thyrotropin-releasing hormone (TRH) promotes wound re-epithelialisation in frog and human skin. PLoS One. 2013;8(9):e73596 10.1371/journal.pone.0073596 . 24023889 PMC3759422

[8] Stojadinovic O, Tomic-Canic M. Human ex vivo wound healing model. Methods Mol Biol. 2013;1037:255–64. Epub 2013/09/14. 10.1007/978-1-62703-505-7_14 . 24029940

[9] Deshayes N, Bloas F, Boissout F, Lecardonnel J, Paris M. 3D In vitro model of the re-epithelialization phase in the wound-healing process. Exp Dermatol. 2018;27(5):460–2. Epub 2017/06/13. 10.1111/exd.13390 . 28603853

[10] Eming SA, Tomic-Canic M. Updates in wound healing: Mechanisms and translation. Exp Dermatol. 2017;26(2):97–8. 10.1111/exd.13281 . 28133858

[11] Garcin CL, Ansell DM. The battle of the bulge: re-evaluating hair follicle stem cells in wound repair. Exp Dermatol. 2017;26(2):101–4. Epub 2016/08/31. 10.1111/exd.13184 . 27574799

[12] Bekeschus S, Schmidt A, Napp M, Kramer A, Kerner W, von Woedtke T, et al Distinct cytokine and chemokine patterns in chronic diabetic ulcers and acute wounds. Exp Dermatol. 2017;26(2):145–7. Epub 2016/09/28. 10.1111/exd.13215 . 27673417

[13] Lebonvallet N, Laverdet B, Misery L, Desmouliere A, Girard D. New insights into the roles of myofibroblasts and innervation during skin healing and innovative therapies to improve scar innervation. Exp Dermatol. 2018;27(9):950–8. Epub 2018/05/10. 10.1111/exd.13681 . 29742295

[14] Silva WN, Prazeres P, Paiva AE, Lousado L, Turquetti AOM, Barreto RSN, et al Macrophage-derived GPNMB accelerates skin healing. Exp Dermatol. 2018;27(6):630–5. Epub 2018/03/06. 10.1111/exd.13524 . 29505115 PMC6013359

[15] Choi EW, Seo MK, Woo EY, Kim SH, Park EJ, Kim S. Exosomes from human adipose-derived stem cells promote proliferation and migration of skin fibroblasts. Exp Dermatol. 2018;27(10):1170–2. Epub 2017/09/25. 10.1111/exd.13451 . 28940813

[16] Harn HI, Ogawa R, Hsu CK, Hughes MW, Tang MJ, Chuong CM. The tension biology of wound healing. Exp Dermatol. 2017 Epub 2017/11/07. 10.1111/exd.13460 . 29105155

[17] Paus R. Exploring the "thyroid-skin connection": concepts, questions, and clinical relevance. J Invest Dermatol. 2010;130(1):7–10. 10.1038/jid.2009.359 . 20010860

[18] Freinkel RK, Freinkel N. Hair growth and alopecia in hypothyroidism. Arch Dermatol. 1972;106(3):349–52. . 5055094

[19] van Beek N, Bodo E, Kromminga A, Gaspar E, Meyer K, Zmijewski MA, et al Thyroid hormones directly alter human hair follicle functions: anagen prolongation and stimulation of both hair matrix keratinocyte proliferation and hair pigmentation. J Clin Endocrinol Metab. 2008;93(11):4381–8. 10.1210/jc.2008-0283 . 18728176

[20] Kaplan MM, Pan CY, Gordon PR, Lee JK, Gilchrest BA. Human epidermal keratinocytes in culture convert thyroxine to 3,5,3'-triiodothyronine by type II iodothyronine deiodination: a novel endocrine function of the skin. J Clin Endocrinol Metab. 1988;66(4):815–22. 10.1210/jcem-66-4-815 . 2450104

[21] Messenger AG. Thyroid hormone and hair growth. Br J Dermatol. 2000;142(4):633–4. . 10792210 10.1046/j.1365-2133.2000.03521.x

[22] Safer JD, Fraser LM, Ray S, Holick MF. Topical triiodothyronine stimulates epidermal proliferation, dermal thickening, and hair growth in mice and rats. Thyroid. 2001;11(8):717–24. 10.1089/10507250152484547 . 11525263

[23] Bodo E, Kany B, Gaspar E, Knuver J, Kromminga A, Ramot Y, et al Thyroid-stimulating hormone, a novel, locally produced modulator of human epidermal functions, is regulated by thyrotropin-releasing hormone and thyroid hormones. Endocrinology. 2010;151(4):1633–42. 10.1210/en.2009-0306 . 20176727

[24] Bodo E, Kromminga A, Biro T, Borbiro I, Gaspar E, Zmijewski MA, et al Human female hair follicles are a direct, nonclassical target for thyroid-stimulating hormone. J Invest Dermatol. 2009;129(5):1126–39. 10.1038/jid.2008.361 . 19052559

[25] Olah A, Gherardini J, Bertolini M, Cheret J, Ponce L, Kloepper J, et al The Thyroid Hormone Analogue KB2115 (Eprotirome) Prolongs Human Hair Growth (Anagen) Ex Vivo. J Invest Dermatol. 2016;136(8):1711–4. 10.1016/j.jid.2016.03.033 . 27066887

[26] Vidali S, Cheret J, Giesen M, Haeger S, Alam M, Watson REB, et al Thyroid Hormones Enhance Mitochondrial Function in Human Epidermis. J Invest Dermatol. 2016;136(10):2003–12. 10.1016/j.jid.2016.05.118 . 27349864

[27] Vidali S, Knuever J, Lerchner J, Giesen M, Biro T, Klinger M, et al Hypothalamic-pituitary-thyroid axis hormones stimulate mitochondrial function and biogenesis in human hair follicles. J Invest Dermatol. 2014;134(1):33–42. 10.1038/jid.2013.286 . 23949722

[28] Holt PJ. In vitro responses of the epidermis to triiodothyronine. J Invest Dermatol. 1978;71(3):202–4. . 690484 10.1111/1523-1747.ep12547158

[29] Holt PJ, Marks R. The epidermal response to change in thyroid status. J Invest Dermatol. 1977;68(5):299–301. . 870564 10.1111/1523-1747.ep12494564

[30] Paus R, Griffiths Ce, Barker JMDe, Bleiker Te, Chalmers Re, Creamer De, et al Rook’s Textbook of dermatology. Ninth edition ed2017 p. 149.1–23.

[31] Erdogan M, Ilhan YS, Akkus MA, Caboglu SA, Ozercan I, Ilhan N, et al Effects of L-thyroxine and zinc therapy on wound healing in hypothyroid rats. Acta Chir Belg. 1999;99(2):72–7. . 10352736

[32] Safer JD, Crawford TM, Holick MF. Topical thyroid hormone accelerates wound healing in mice. Endocrinology. 2005;146(10):4425–30. 10.1210/en.2005-0192 . 15976059

[33] Kress E, Samarut J, Plateroti M. Thyroid hormones and the control of cell proliferation or cell differentiation: paradox or duality? Mol Cell Endocrinol. 2009;313(1–2):36–49. 10.1016/j.mce.2009.08.028 . 19737599

[34] Nygaard B, Saedder EA, Dalhoff K, Wikkelsoe M, Jurgens G. Levothyroxine Poisoning—Symptoms and Clinical Outcome. Basic Clin Pharmacol Toxicol. 2015;117(4):280–5. Epub 2015/03/31. 10.1111/bcpt.12401 . 25816846

[35] Ito M, Yang Z, Andl T, Cui C, Kim N, Millar SE, et al Wnt-dependent de novo hair follicle regeneration in adult mouse skin after wounding. Nature. 2007;447(7142):316–20. 10.1038/nature05766 . 17507982

[36] Ansell DM, Kloepper JE, Thomason HA, Paus R, Hardman MJ. Exploring the "hair growth-wound healing connection": anagen phase promotes wound re-epithelialization. J Invest Dermatol. 2011;131(2):518–28. 10.1038/jid.2010.291 . 20927125

[37] Jimenez F, Poblet E, Izeta A. Reflections on how wound healing-promoting effects of the hair follicle can be translated into clinical practice. Exp Dermatol. 2015;24(2):91–4. Epub 2014/07/30. 10.1111/exd.12521 . 25066054

[38] Lu Z, Hasse S, Bodo E, Rose C, Funk W, Paus R. Towards the development of a simplified long-term organ culture method for human scalp skin and its appendages under serum-free conditions. Exp Dermatol. 2007;16(1):37–44. 10.1111/j.1600-0625.2006.00510.x . 17181635

[39] Moll I, Houdek P, Schmidt H, Moll R. Characterization of epidermal wound healing in a human skin organ culture model: acceleration by transplanted keratinocytes. J Invest Dermatol. 1998;111(2):251–8. 10.1046/j.1523-1747.1998.00265.x . 9699726

[40] Gurtner GC, Werner S, Barrandon Y, Longaker MT. Wound repair and regeneration. Nature. 2008;453(7193):314–21. 10.1038/nature07039 . 18480812

[41] Raja, Sivamani K, Garcia MS, Isseroff RR. Wound re-epithelialization: modulating keratinocyte migration in wound healing. Front Biosci. 2007;12:2849–68. . 17485264 10.2741/2277

[42] Singer AJ, Clark RA. Cutaneous wound healing. N Engl J Med. 1999;341(10):738–46. 10.1056/NEJM199909023411006 . 10471461

[43] Abramo F, Pirone A, Lenzi C, Vannozzi I, Della Valle MF, Miragliotta V. Establishment of a 2-week canine skin organ culture model and its pharmacological modulation by epidermal growth factor and dexamethasone. Ann Anat. 2016;207:109–17. Epub 2016/04/09. 10.1016/j.aanat.2016.03.009 . 27058637

[44] Mecklenburg L, Tobin DJ, Muller-Rover S, Handjiski B, Wendt G, Peters EM, et al Active hair growth (anagen) is associated with angiogenesis. J Invest Dermatol. 2000;114(5):909–16. 10.1046/j.1523-1747.2000.00954.x . 10771470

[45] Davis FB, Mousa SA, O’Connor L, Mohamed S, Lin HY, Cao HJ, et al Proangiogenic action of thyroid hormone is fibroblast growth factor-dependent and is initiated at the cell surface. Circ Res. 2004;94(11):1500–6. 10.1161/01.RES.0000130784.90237.4a . 15117822

[46] Cao Y, Arbiser J, D’Amato RJ, D’Amore PA, Ingber DE, Kerbel R, et al Forty-year journey of angiogenesis translational research. Sci Transl Med. 2011;3(114):114rv3 10.1126/scitranslmed.3003149 . 22190240 PMC8265598

[47] Choi SM, Lee KM, Kim HJ, Park IK, Kang HJ, Shin HC, et al Effects of structurally stabilized EGF and bFGF on wound healing in type I and type II diabetic mice. Acta Biomater. 2018;66:325–34. Epub 2017/12/06. 10.1016/j.actbio.2017.11.045 . 29203426

[48] Aoki S, Fujii M, Fujie T, Nishiwaki K, Miyazaki H, Saitoh D, et al The efficacy of basic fibroblast growth factor-loaded poly(lactic-co-glycolic acid) nanosheet for mouse wound healing. Wound Repair Regen. 2017;25(6):1008–16. Epub 2018/01/10. 10.1111/wrr.12604 . 29315978

[49] Kinoda J, Ishihara M, Nakamura S, Fujita M, Fukuda K, Sato Y, et al Protective effect of FGF-2 and low-molecular-weight heparin/protamine nanoparticles on radiation-induced healing-impaired wound repair in rats. J Radiat Res. 2018;59(1):27–34. Epub 2017/11/10. 10.1093/jrr/rrx044 . 29121251 PMC5778538

[50] Shamloo A, Sarmadi M, Aghababaie Z, Vossoughi M. Accelerated full-thickness wound healing via sustained bFGF delivery based on a PVA/chitosan/gelatin hydrogel incorporating PCL microspheres. Int J Pharm. 2018;537(1–2):278–89. Epub 2017/12/31. 10.1016/j.ijpharm.2017.12.045 . 29288809

[51] Ogino S, Morimoto N, Sakamoto M, Jinno C, Sakamoto Y, Taira T, et al Efficacy of the dual controlled release of HGF and bFGF impregnated with a collagen/gelatin scaffold. J Surg Res. 2018;221:173–82. Epub 2017/12/13. 10.1016/j.jss.2017.08.051 . 29229125

[52] Saijo H, Kilpadi DV, Akita S. Evaluation of the use of recombinant human basic fibroblast growth factor in combination with negative pressure wound therapy with instillation and dwell time in porcine full-thickness wound model. Wound Repair Regen. 2017;25(6):972–5. Epub 2018/01/13. 10.1111/wrr.12609 . 29328528

[53] Rotty JD, Coulombe PA. A wound-induced keratin inhibits Src activity during keratinocyte migration and tissue repair. J Cell Biol. 2012;197(3):381–9. 10.1083/jcb.201107078 . 22529101 PMC3341159

[54] Wong P, Coulombe PA. Loss of keratin 6 (K6) proteins reveals a function for intermediate filaments during wound repair. J Cell Biol. 2003;163(2):327–37. Epub 2003/10/22. 10.1083/jcb.200305032 . 14568992 PMC2173512

[55] Philpott MP, Green MR, Kealey T. Human hair growth in vitro. J Cell Sci. 1990;97 (Pt 3):463–71. . 1705941 10.1242/jcs.97.3.463

[56] Bodo E, Kromminga A, Funk W, Laugsch M, Duske U, Jelkmann W, et al Human hair follicles are an extrarenal source and a nonhematopoietic target of erythropoietin. FASEB J. 2007;21(12):3346–54. 10.1096/fj.07-8628com . 17540710

[57] Bodo E, Tobin DJ, Kamenisch Y, Biro T, Berneburg M, Funk W, et al Dissecting the impact of chemotherapy on the human hair follicle: a pragmatic in vitro assay for studying the pathogenesis and potential management of hair follicle dystrophy. Am J Pathol. 2007;171(4):1153–67. 10.2353/ajpath.2007.061164 . 17823286 PMC1988866

[58] Telek A, Biro T, Bodo E, Toth BI, Borbiro I, Kunos G, et al Inhibition of human hair follicle growth by endo- and exocannabinoids. FASEB J. 2007;21(13):3534–41. 10.1096/fj.06-7689com . 17567570

[59] Arbiser JL, Byers HR, Cohen C, Arbeit J. Altered basic fibroblast growth factor expression in common epidermal neoplasms: examination with in situ hybridization and immunohistochemistry. J Am Acad Dermatol. 2000;42(6):973–7. . 10827398

[60] Knuever J, Poeggeler B, Gaspar E, Klinger M, Hellwig-Burgel T, Hardenbicker C, et al Thyrotropin-releasing hormone controls mitochondrial biology in human epidermis. J Clin Endocrinol Metab. 2012;97(3):978–86. 10.1210/jc.2011-1096 . 22259067

[61] Poeggeler B, Knuever J, Gaspar E, Biro T, Klinger M, Bodo E, et al Thyrotropin powers human mitochondria. FASEB J. 2010;24(5):1525–31. 10.1096/fj.09-147728 . 20075194

[62] Stenn KS. Epibolin: a protein of human plasma that supports epithelial cell movement. Proc Natl Acad Sci U S A. 1981;78(11):6907–11. . 6171824 10.1073/pnas.78.11.6907 PMC349161

[63] Wojcik SM, Bundman DS, Roop DR. Delayed wound healing in keratin 6a knockout mice. Mol Cell Biol. 2000;20(14):5248–55. . 10866680 10.1128/mcb.20.14.5248-5255.2000 PMC85973

[64] Luidens MK, Mousa SA, Davis FB, Lin HY, Davis PJ. Thyroid hormone and angiogenesis. Vascul Pharmacol. 2010;52(3–4):142–5. 10.1016/j.vph.2009.10.007 . 19879961

[65] Martinez ML, Escario E, Poblet E, Sanchez D, Buchon FF, Izeta A, et al Hair follicle-containing punch grafts accelerate chronic ulcer healing: A randomized controlled trial. J Am Acad Dermatol. 2016;75(5):1007–14. 10.1016/j.jaad.2016.02.1161 . 27745629

[66] Poblet E, Jimenez F, Escario-Travesedo E, Hardman JA, Hernandez-Hernandez I, Agudo-Mena JL, et al Eccrine sweat glands associate with the human hair follicle within a defined compartment of dermal white adipose tissue. Br J Dermatol. 2018;178(5):1163–72. Epub 2018/02/13. 10.1111/bjd.16436 . 29432654

[67] Cheret J, Bertolini M, Ponce L, Lehmann J, Tsai T, Alam M, et al Olfactory receptor OR2AT4 regulates human hair growth. Nat Commun. 2018;9(1):3624 Epub 2018/09/20. 10.1038/s41467-018-05973-0 . 30228264 PMC6143528

[68] Hawkshaw NJ, Hardman JA, Haslam IS, Shahmalak A, Gilhar A, Lim X, et al Identifying novel strategies for treating human hair loss disorders: Cyclosporine A suppresses the Wnt inhibitor, SFRP1, in the dermal papilla of human scalp hair follicles. PLoS Biol. 2018;16(5):e2003705 Epub 2018/05/09. 10.1371/journal.pbio.2003705 . 29738529 PMC5940179

[69] Samuelov L, Sprecher E, Tsuruta D, Biro T, Kloepper JE, Paus R. P-cadherin regulates human hair growth and cycling via canonical Wnt signaling and transforming growth factor-beta2. J Invest Dermatol. 2012;132(10):2332–41. Epub 2012/06/15. 10.1038/jid.2012.171 . 22696062

[70] Wen J, Li X, Leng X, Xu X, Wu X. An advanced mouse model for human skin wound healing. Exp Dermatol. 2017;26(5):433–5. Epub 2016/11/29. 10.1111/exd.13258 . 27892608

[71] Certan D, Righini V, Oliva M, Fioravanti P, Bevilacqua M. Bioavailability of l-thyroxine and its metabolites after topical treatment with an emulsion containing 0.1% micronised l-thyroxine. G Ital Dermatol Venereol. 2013;148(3):287–92. . 23670065

[72] Orlandi C, Bondioli E, Venturi M, Melandri D. Preliminary observations of a new approach to tissue repair: Peripheral blood mononuclear cells in platelet-rich plasma injected into skin graft area. Exp Dermatol. 2018;27(7):795–7. Epub 2018/04/01. 10.1111/exd.13552 . 29604139

[73] Takagi N, Kawakami K, Kanno E, Tanno H, Takeda A, Ishii K, et al IL-17A promotes neutrophilic inflammation and disturbs acute wound healing in skin. Exp Dermatol. 2017;26(2):137–44. Epub 2016/06/16. 10.1111/exd.13115 . 27305096

[74] Mizukami Y, Sugawara K, Kira Y, Tsuruta D. Sorafenib stimulates human skin type mast cell degranulation and maturation. J Dermatol Sci. 2017;88(3):308–19. Epub 2017/08/28. 10.1016/j.jdermsci.2017.08.005 . 28843624

[75] Cappellano G, Morandi EM, Rainer J, Grubwieser P, Heinz K, Wolfram D, et al Human Macrophages Preferentially Infiltrate the Superficial Adipose Tissue. Int J Mol Sci. 2018;19(5). Epub 2018/05/09. 10.3390/ijms19051404 . 29738484 PMC5983635

[76] Sugawara K, Biro T, Tsuruta D, Toth BI, Kromminga A, Zakany N, et al Endocannabinoids limit excessive mast cell maturation and activation in human skin. J Allergy Clin Immunol. 2012;129(3):726–38 e8. Epub 2012/01/10. 10.1016/j.jaci.2011.11.009 . 22226549

[77] Abramo F, Lazzarini G, Pirone A, Lenzi C, Albertini S, Della Valle MF, et al Ultramicronized palmitoylethanolamide counteracts the effects of compound 48/80 in a canine skin organ culture model. Vet Dermatol. 2017;28(5):456–e104. Epub 2017/06/07. 10.1111/vde.12456 . 28585337

[78] Markova A, Mostow EN. US skin disease assessment: ulcer and wound care. Dermatol Clin. 2012;30(1):107–11, ix 10.1016/j.det.2011.08.005 . 22117872

[79] Poumay Y, Roland IH, Leclercq-Smekens M, Leloup R. Basal detachment of the epidermis using dispase: tissue spatial organization and fate of integrin alpha 6 beta 4 and hemidesmosomes. J Invest Dermatol. 1994;102(1):111–7. . 8288902 10.1111/1523-1747.ep12371742

